# Inflammation Is Present, Persistent and More Sensitive to Proinflammatory Triggers in Celiac Disease Enterocytes

**DOI:** 10.3390/ijms23041973

**Published:** 2022-02-10

**Authors:** Monia Porpora, Mariangela Conte, Giuliana Lania, Claudia Bellomo, Luciano Rapacciuolo, Fernando Gabriel Chirdo, Renata Auricchio, Riccardo Troncone, Salvatore Auricchio, Maria Vittoria Barone, Merlin Nanayakkara

**Affiliations:** 1 ELFID (European Laboratory for the Investigation of Food Induced Diseases), Department of Translational Medical Science, Section of Paediatrics, University Federico II, Via S. Pansini 5, 80131 Naples, Italy; monia.porpora@hotmail.it (M.P.); maryconte_92@hotmail.it (M.C.); giuliana.lania@gmail.com (G.L.); bellomo.claudia96@gmail.com (C.B.); lrapacci@unina.it (L.R.); r.auricchio@unina.it (R.A.); troncone@unina.it (R.T.); salauric@unina.it (S.A.); merlinnanayakkara@gmail.com (M.N.); 2 Departamento de Ciencias Biologicas, Facultad de Ciencias Exactas, Instituto de Estudios Inmunologicos y Fisiopatologicos—IIFP (UNLP-CONICET), Bv. 120 1489, La Plata 1900, Argentina; fchirdo@biol.unlp.edu.ar

**Keywords:** small intestine, potential celiac disease, NF-κB, ERK

## Abstract

Celiac disease (CD) is a chronic inflammatory disease caused by a genetic predisposition to an abnormal T cell-mediated immune response to the gluten in the diet. Different environmental proinflammatory factors can influence and amplify the T cell-mediated response to gluten. The aim of this manuscript was to study the role of enterocytes in CD intestinal inflammation and their response to different proinflammatory factors, such as gliadin and viruses. Intestinal biopsies from CD patients on a gluten-containing (GCD-CD) or a gluten-free diet (GFD-CD) as well as biopsies from potential CD patients (Pot-CD) before the onset of intestinal lesions and controls (CTR) were used to investigate IL-1β and IL-6 mRNA levels in situ. Organoids from CD patients were used to test the levels of NF-κB, ERK, IL-6, and IL-1β by Western blot (WB), ELISA, and quantitative PCR. The Toll-like receptor ligand loxoribine (Lox) and gliadin peptide P31-43 were used as proinflammatory stimuli. In CD biopsies inflammation markers IL-1β and IL-6 were increased in the enterocytes, and also in Pot-CD before the onset of the intestinal lesion and in GFD-CD. The inflammatory markers pNF-κB, pERK, IL-1β, and IL-6 were increased and persistent in CD organoids; these organoids were more sensitive to P31-43 and Lox stimuli compared with CTR organoids. Taken together, these observations point to constitutive inflammation in CD enterocytes, which are more sensitive to inflammatory stimuli such as food components and viruses.

## 1. Introduction

Celiac disease (CD) is an immune-mediated enteropathy triggered in genetically susceptible individuals by a group of wheat proteins and related prolamins from cereals [1]. The HLA-restricted gliadin-specific intestinal T cell response plays a central role in the pathogenesis of CD [1]. Moreover, CD is associated with various extra-intestinal diseases including several skin manifestations [2].

CD is known to be characterized by a combination of gluten-induced symptoms, the generation of CD-associated autoantibodies, and enteropathy [3], but it remains unclear why T cells are activated by gliadin. Studies conducted in mice have demonstrated that mucosal inflammation due to reovirus infection may disrupt oral tolerance to gliadin by suppressing regulatory T cell conversion and promoting Th1 immunity [4]. These results indicate that in an inflamed environment enriched in cytokines, T cells tend to acquire a proinflammatory phenotype. The factors that create a proinflammatory environment in the CD intestine could have multiple origins. Recent studies have described the influence of several different factors in CD, such as cellular vulnerability, the proinflammatory effects of gluten [5,6] and other wheat proteins [7], Western diet [8] and other environmental triggers, such as viruses [4,9], which may prepare and/or amplify the TC-mediated response to gluten [10].

Cellular vulnerability in CD has been suggested by recent studies that have shown that despite clinical and histological remission, celiac disease patients fed a gluten-free diet (GFD) have altered protein composition of gut tissue with signs of ongoing inflammation. In fact, changes indicative of epithelial inflammation, minor crypt hyperplasia, and low-grade inflammation in the serum have been demonstrated in GFD-CD intestinal biopsies before gluten challenge in vivo [11]. Moreover, alterations in differentiation/proliferation pathways have been found in GFD-CD patients before in vivo challenge with gluten [12]. Interestingly, after in vivo challenge with gluten, an increase in the same proteins that were altered at baseline in the GFD-CD was found [11,12]. Nuclear factor kappa-light-chain-enhancer of activated B cells (NF-κB), a major mediator of the inflammatory response [13], has been found to be increased in GCD-CD and GFD-CD biopsies from several different genetic [14] and expression studies [15,16,17]. Fernandez-Jimenez et al. [15] showed that the expression of NF-κB genes measured by RT–PCR (real-time polymerase chain reaction) was altered in GFD-CD and GCD-CD compared to control biopsies and in cultured biopsies from CD (GCD and GFD) patients challenged with gliadin. These results showed that genes that were constitutively upregulated in GFD patients belonged to the NF-κB signaling system. Interestingly, according to GFD biopsies, gluten challenge also increased NF-κB pathway activation in vitro. Similar increases in the activation of the NF-κB pathway were observed in GFD-CD biopsies and fibroblasts, both derived from the intestines of CD patients and from skin explants (distant from the intestine). Only biopsies and fibroblasts from CD patients were sensitive to low doses of gliadin peptide P31-43, which did not affect controls [18]. Taken together, these data indicate that in CD, a vulnerability (probably constitutive) is present that renders cells more sensitive to proinflammatory stimuli.

The intestinal epithelium has assumed an emerging role in CD pathogenesis; in fact, morphological [5,18] and functional alterations [18,19] have been described in epithelial cells in CD patients. Moreover, the inflammasome pathway was increased in intestinal epithelial cells isolated from GFD-CD biopsies after gluten challenge [20]. Moreover, IL-1beta and IL-6 are part of the NF-κB pathway [21,22] and are important mediators of the inflammation at the level of the intestine [23].

Gluten and, in particular, one of its undigested peptides (P31-43) can induce inflammation in cultured cells, in CD biopsies (GCD and GFD), and in mice [6]. P31-43 is not presented to T cells and can induce several different effects on cells, in CD biopsies and mice, including proliferation, activation of innate immune markers, and inflammation [6,24]. Moreover, it can act synergistically with viral ligands (Poli I:C and loxoribine) to induce innate immunity activation both in vitro and in vivo [25]

Therefore, the emerging role of epithelial inflammation and the proinflammatory effects of viruses and gliadin in CD prompted us to study inflammatory markers in situ in the enterocytes of biopsies derived from CD patients at different stages of the disease (GCD-, GFD-, and Pot-CD). Moreover, we examined whether intestinal organoids derived from CD biopsies are a good model for studying inflammation and sensitivity to proinflammatory agents, such as P31-43 and the viral ligand loxoribine, in CD epithelium.

## 2. Results

### 2.1. In CD Biopsies, the Inflammatory Markers IL-1β and IL-6 Are Increased in Enterocytes

**Figure 1:**
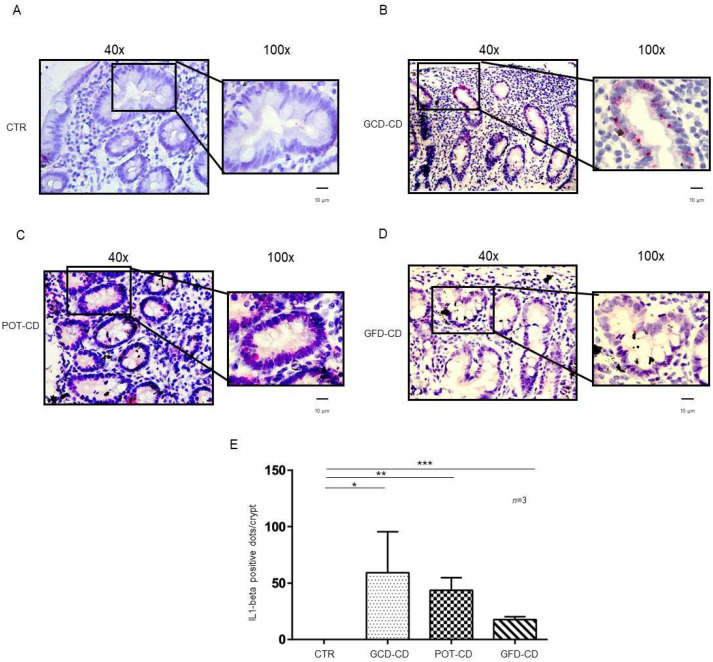
L-1β is increased in the epithelium of the crypts in CD biopsies: In situ mRNA analysis of IL-1βeta in biopsies from controls (CTR) (**A**), gluten-containing diet celiac disease patients (GCD-CD) (**B**), Potential CD patients (Pot-CD) (**C**), and gluten-free diet celiac patients (GFD-CD) (**D**). Blue indicates hematoxylin–eosin staining of the nuclei, and red indicates IL-1β mRNA. Black squares show different enlargements of crypts. Lines indicate 10 micrometers at 100× objective. (**E**). IL-1β-positive red dot counts in the crypts of intestinal biopsies from CD patients and controls. At least 10 crypts/subject were counted on different slides. The numbers of subjects analyzed are indicated. Columns represent the mean, and bars represent the standard deviation. Student’s *t* test: * = *p* < 0.05; ** = *p* < 0.01; *** = *p* < 0.001.

IL-1β and IL-6 are part of the NF-κB pathway [21,22], they are important mediators of the inflammation at the level of the intestine [23] and have been related to inflammatory bowel diseases [26,27]. Expression of IL-1β and IL-6 mRNA in situ was analyzed in intestinal biopsies from CD patients at different stages of the disease as well as those from controls. IL-1β mRNA was absent in control biopsies at both the level of the intestinal epithelial cells of the crypts (Figure 1A,E) and of the villi (Supplemental Figure S1A). In contrast, IL-1β mRNA was increased in all CD patients independent of the stage of the disease and almost exclusively at the level of the crypts (Figure 1B–D; Supplemental Figure S1B–E). In particular, IL-1β mRNA was highly expressed (59 ± 36 red dots/crypt, *p* value < 0.05) in CD patients fed a gluten-containing diet (GCD-CD) (Figure 1B,E) in the acute phase of the disease and in potential patients (Pot-CD) (43.6 ± 11 red dots/crypt, *p* value < 0.01) (Figure 1C,E) before the onset of lesions of the small intestine, who still consumed gluten. Patients in the remission phase of the disease (GFD-CD) on a gluten-free diet also presented an increased expression of IL-1β in the intestinal epithelium, although at lower levels, than patients in the GCD-CD and Pot-CD groups (17.6 ± 2.5 red dots/crypt, *p* value < 0.001) (Figure 1D,E).

**Figure 2:**
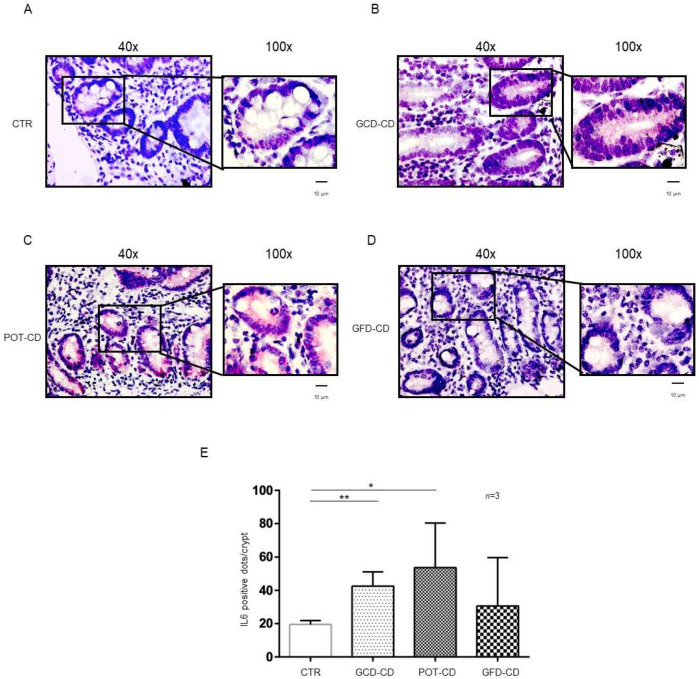
IL-6 levels were increased in the epithelium of the crypts in CD biopsies: In situ mRNA analysis of IL-6 in biopsies from controls (CTR) (**A**), gluten-containing diet celiac disease patients (GCD-CD) (**B**), Potential CD patients (Pot-CD) (**C**), and gluten-free diet celiac patients (GFD-CD) (**D**). Blue indicates hematoxylin–eosin staining of the nuclei, and red indicates IL-6 mRNA. Black squares show different enlargements of crypts. Lines indicate 10 micrometers at 100× objective. (**E**). IL-6-positive red dot counts in the crypts of intestinal biopsies from CD patients and controls. At least 10 crypts/subject were counted on different slides. The numbers of subjects analyzed are indicated. Columns represent the mean, and bars represent the standard deviation. Student’s *t* test: * = *p* < 0.05; ** = *p* < 0.01.

IL-6 mRNA was present in control biopsies both at the level of the crypts (Figure 2A,E) and of the villi (Supplemental Figure S2A), as expected [28]. On the other hand, IL-6, similar to IL-1β (Figure 2), was overexpressed with respect to the control in the GCD-CD, GFD-CD, and Pot-CD groups (Figure 2B–D).

In particular, IL-6 mRNA was highly expressed (42.5 ± 8.6 red dots/crypt) in CD patients fed a gluten-containing diet (GCD-CD) (Figure 2B,E; *p* value < 0.01) in the acute phase of the disease and in potential patients (Pot-CD) (53.67 ± 26.7 red dots/crypt; *p* value < 0.05) (Figure 2C,E) before the onset of lesions of the small intestine, who still consumed gluten. Patients in the remission phase of the disease and fed a gluten-free diet (GFD-CD) (Figure 2D) also presented altered expression of IL-6 in the intestinal epithelium; although, the difference did not reach statistical significance (30.67 ± 29.01 red dots/crypt) (Figure 2D,E).

### 2.2. The Inflammatory Markers pNF-κB, pERK, IL-1β, and IL-6 Were Increased and Persistent in CD Organoids

**Figure 3:**
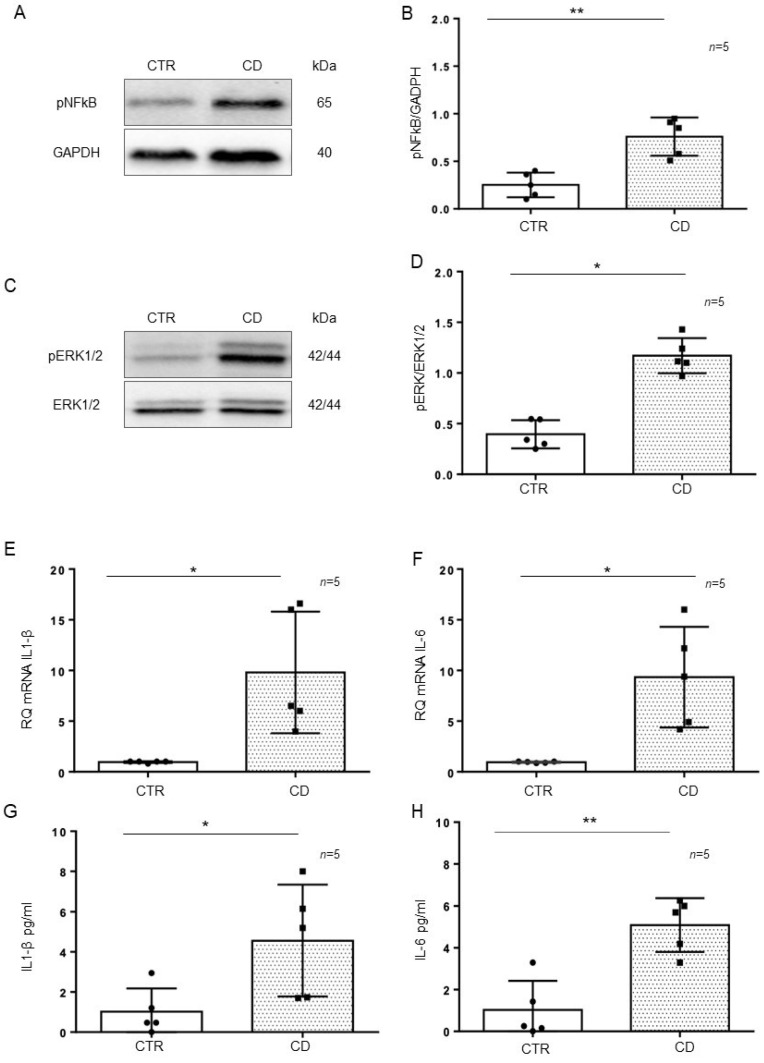
Inflammatory markers were increased in CD organoids: (**A**). Western blot analysis of total protein lysates of organoids from controls (CTR) and CD patients fed a gluten-containing diet (CD). Upper lines were blotted with an antibody against the phosphorylated form of NF-κB (pNF-κB). Bottom lines were blotted with anti-GAPDH (glyceraldehyde-3-phosphate dehydrogenase) antibody as a loading control. Representative images were selected. (**B**). Densitometric analysis of the pNF-κB/GAPDH bands from CTR and CD. The numbers of organoids analyzed are indicated. Columns represent the mean, and bars represent the standard deviation. Student’s t test: ** = *p* < 0.01. (**C**). Western blot analysis of total protein lysates of organoids from CTR and CD. Upper lines were blotted with an antibody against the phosphorylated form of ERK (pERK). Bottom lines were blotted with anti-ERK antibody as a loading control. Representative images were selected. (**D**). Densitometric analysis of the pERK/ERK bands from CTR and CD. The numbers of organoids analyzed are indicated. Columns represent the mean, and bars represent the standard deviation. Student’s t test: * = *p* < 0.05. (**E**,**F**). Quantitative PCR analysis of IL-1β and IL-6 mRNA levels in CD patients compared to CTR patients. The number of organoids analyzed is indicated. The columns represent the mean, and bars represent the standard deviation. Student’s *t* test: * = *p* < 0.05. (**G**,**H**). ELISA showing IL-1β and IL-6 protein levels in the culture media of CD organoids compared to CTR organoids. The numbers of organoids are indicated. The columns represent the mean, and bars represent the standard deviation. Student’s t test: * = *p* < 0.05; ** = *p* < 0.01.

Intestinal organoids were derived from GCD-CD and CTR intestinal biopsies and tested after 4 weeks of culture for the inflammatory markers pNF-κB, pERK, IL-1β, and IL-6. In particular, pNF-κB (*p* value < 0.01) and pERK (*p* value < 0.05) were increased in CD organoids compared to CTR organoids, as evaluated by Western blot (WB) analysis (Figure 3A–D). Quantitative mRNA (Figure 3E,F) showed a marked increase in IL-1β (*p* value < 0.05) and IL-6 (*p* value < 0.05) expression in CD organoids compared with that of CTR organoids. An ELISA of the supernatant of the organoid cultures (Figure 3G,H) showed a marked increase in IL-1β (*p* value < 0.05) and IL-6 (*p* value < 0.01) levels in the CD supernatant compared with those of CTR supernatant.

**Figure 4:**
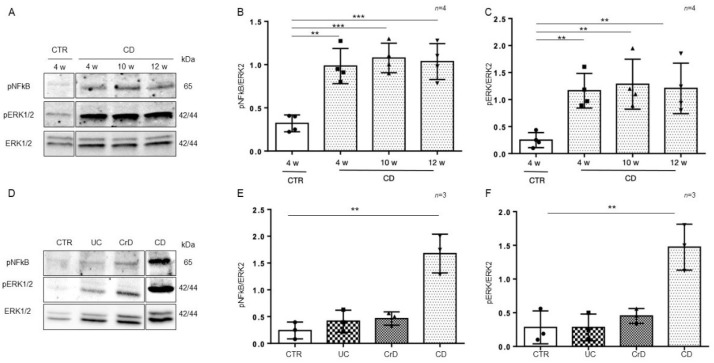
Inflammation was persistent in CD organoids: (**A**). Western blot analysis of the total protein lysates of organoids from CD patients cultivated for several weeks as indicated. Upper lines were blotted with an antibody against the phosphorylated form of NF-κB (pNF-κB). Middle lines were blotted with an antibody against the phosphorylated form of ERK (pERK). Bottom lines were blotted with anti-ERK antibody as a loading control. Representative images were selected. (**B**). Densitometric analysis of the pNF-κB/ERK bands from CD cultivated for different times as indicated. The numbers of subjects analyzed are indicated. Columns represent the mean, and bars represent the standard deviation. Student’s *t* test: ** = *p* < 0.01; *** = *p* < 0.001. (**C**). Densitometric analysis of the pERK/ERK bands from CD cultivated for different times as indicated. The numbers of subjects analyzed are indicated. Columns represent the mean, and bars represent the standard deviation. Student’s *t* test: ** = *p* < 0.01. (**D**). Western blot analysis of total protein lysates of organoids from CTR, ulcerative colitis (UC), Crohn’s disease (CrD), and CD cultivated for 4 weeks. Upper lines were blotted with an antibody against the phosphorylated form of NF-κB (pNF-κB). Middle lines were blotted with an antibody against the phosphorylated form of ERK (pERK). Bottom lines were blotted with anti-ERK antibody as a loading control. Representative images were selected. (**E**). Densitometric analysis of the pNF-κB/ERK bands from CTR, UC, CrD, and CD cultivated for 4 weeks. The numbers of subjects analyzed are indicated. Columns represent the mean, and bars represent the standard deviation. Student’s *t* test: ** = *p* < 0.01. (**F**). Densitometric analysis of the pERK/ERK bands from CTR, UC, CrD, and CD cultivated for 4 weeks. The numbers of subjects analyzed are indicated. Columns represent the mean, and bars represent the standard deviation. Student’s *t* test: ** = *p* < 0.01.

To understand whether the inflammation in intestinal organoids was a residual effect of the inflamed intestinal environment or inherent to the intestinal CD epithelium, we cultivated organoids from CD and CTR patients for several weeks (from 4 to 12 weeks) and then tested them again for the inflammatory markers pNF-κB and pERK by WB analysis (Figure 4A–C). Interestingly, the expression of the inflammatory markers pNF-κB (*p* value < 0.01, < 0.001) and pERK (*p* value < 0.01) in CD organoids remained more elevated than in CTR organoids after several weeks in culture. 

In intestinal organoids from inflammatory bowel disease (IBD) patients, markers of inflammation are elevated only for a few weeks (1–2 w) after culture and then decrease to the level of CTR organoids [29]. In Figure 4D–F, we compared the inflammatory markers pNF-κB and pERK in intestinal organoids from CD and IBD patients after 4 weeks in culture. We confirmed that in IBD, both pNF-κB and pERK were at the level of the controls. Only CD organoids showed higher levels of pNF-κB (*p* value < 0.01) and pERK (*p* value < 0.01), than the CTR organoids after 4 weeks in culture. Taken together, this indicates that in CD, contrary to IBD, inflammation is persistent.

### 2.3. 3D and 2D Organoids from CD Patients Had Increased Inflammatory Markers Compared to Those from CTR Patients

Organoids are considered good models for studying inflammation and infection of the intestine [30].

**Figure 5:**
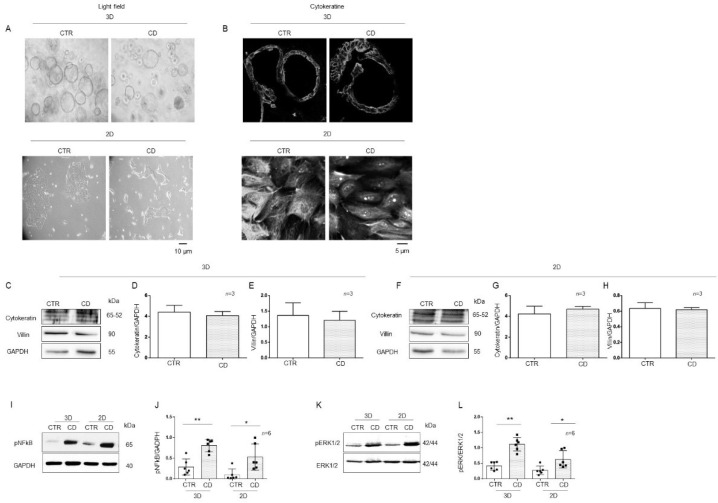
Markers of inflammation were increased in both 3D and 2D organoids from CD patients compared to CTR patients: (**A**). Light field microscopy of 3D and 2D organoids from CD and CTR. 20X objective. The black line indicates 10 microns. (**B**) Immunofluorescence images of 3D and 2D organoids stained with anti-cytokeratin antibodies. 60× objective. The black line indicates 5 microns. (**C**). Western blot analysis of total protein lysates from CTR and CD 3D organoids. Upper lines were blotted with an antibody against cytokeratin. Middle lines were blotted with an antibody against villin. Bottom lines were blotted with GAPDH antibody as a loading control. Representative images were selected. Densitometric analysis of the cytokeratine/GAPDH (**D**) and villin/GAPDH (**E**) bands from 3D organoids from CTR and CD. Columns represent the mean, and bars represent the standard deviations. The numbers of subjects analyzed are indicated. (**F**). Western blot analysis of total protein lysates from CTR and CD 2D organoids. Upper lines were blotted with an antibody against cytokeratin. Middle lines were blotted with an antibody against villin. Bottom lines were blotted with GAPDH antibody as a loading control. Representative images were selected. Densitometric analysis of the cytokeratin/GAPDH (**G**) and villin/GAPDH (**H**) bands from 2D organoids from CTR and CD. Columns represent the mean, and bars represent the standard deviations. The numbers of subjects analyzed are indicated. (**I**). Western blot analysis of total protein lysates of 3D and 2D organoids from CTR and CD patients. Upper lines were blotted with an antibody against the phosphorylated form of NF-κB (pNF-κB). Lower lines were blotted with anti-GAPDH antibody as a loading control. Representative images were selected. (**J**). Densitometric analysis of the pNF-κB/GAPDH bands from 3D and 2D organoids from CTR and CD. The numbers of organoids analyzed are indicated. Columns represent the mean, and bars represent the standard deviation. Student’s *t* test: * = *p* < 0.05; ** = *p* < 0.01. (**K**). Western blot analysis of total protein lysates of 3D and 2D organoids from CTR and CD. Upper lines were blotted with an antibody against the phosphorylated form of ERK (pERK). Bottom lines were blotted with anti-ERK antibody as a loading control. Representative images were selected. (**L**). Densitometric analysis of the pERK/ERK bands from CTR and CD. The numbers of organoids analyzed are indicated. Columns represent the mean, and bars represent the standard deviation. Student’s *t* test: * = *p* < 0.05; ** = *p* < 0.01.

To provide correct treatment for the apical side of the intestinal cells of organoids, it is necessary to open them up, shifting them to 2D because in 3D, the apical side of the cells is enclosed in the spherical organoids. Light microscopy analysis of organoids in 3D and 2D did not show any difference in their dimensions. Three-dimensional CD organoids were denser than CTR organoids, as already described [31] (Figure 5A). The expression of villin and cytokeratin, differentiation markers of epithelial cells, was similar in 3D and 2D organoids, as assessed by immunofluorescence and WB (Figure 5B–H). Phosphorylation levels of NF-κB and ERK in 3D organoids were compared to those in 2D organoids, for both CD and CTR patients (Figure 5I–L). pNF-κB was increased in both 2D (*p* value < 0.05) and 3D (*p* value < 0.01) CD organoids compared to CTR organoids. Additionally, pERK was increased in both 2D (*p* value < 0.05) and 3D (*p* value < 0.01) CD organoids compared to CTR organoids. Moreover, IL-1β and IL-6 measured by RT PCR and ELISA were increased in CD organoids respect to controls as shown in Supplemental Figure S3. We confirmed that opening the organoids did not affect inflammatory marker expression (Figure 5I–L).

### 2.4. Organoids from CD Patients Were More Sensitive to P31-43

**Figure 6:**
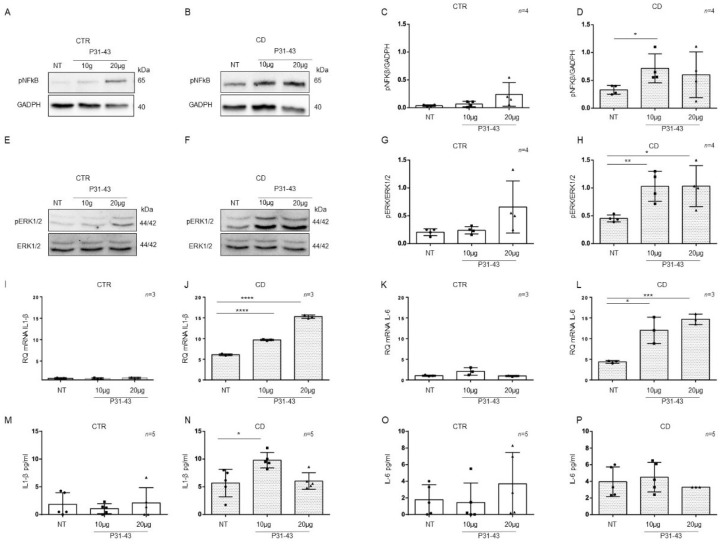
Organoids from CD patients, but not from CTR patients, are inflamed by P31-43: Western blot analysis of total protein lysates of organoids from CTR (**A**) and CD (**B**) before (NT) and after treatment with low concentrations of P31-43 10 μg/mL (10 μg) and 20 μg/mL (20 μg). Upper lines were blotted with an antibody against the phosphorylated form of NF-κB (pNF-κB). Bottom lines were blotted with anti-GAPDH antibodies as a loading control. Representative images were selected. (**C**,**D**). Densitometric analysis of the pNF-κB/GAPDH bands from CTR and CD before and after treatment with P31-43 as indicated. The numbers of organoids analyzed are indicated. Columns represent the mean, and bars represent the standard deviations. Student’s t test: * = *p* < 0.05. (**E**,**F**). Western blot analysis of total protein lysates of organoids from CTR (**E**) and CD (**F**) patients before (NT) and after treatment with P31-43 as indicated. Upper lines were blotted with an antibody against the phosphorylated form of ERK (pERK). Bottom lines were blotted with anti-ERK antibody as a loading control. Representative images were selected. (**G**,**H**). Densitometric analysis of the pERK/ERK bands from CTR and CD before and after treatment with P31-43 as indicated. The numbers of subjects analyzed are indicated. Columns represent the mean, and bars represent the standard deviation. Student’s t test: * = *p* < 0.05; ** = *p* < 0.01. (**I**,**J**). Quantitative PCR analysis of IL-1β mRNA levels in organoids from CD patients compared to CTR organoids. The numbers of subjects analyzed are indicated. Student’s t test: **** = *p* < 0.0001. (**K**,**L**). Quantitative PCR analysis of IL-6 mRNA levels in organoids from CD patients compared to CTR organoids. The numbers of organoids analyzed are indicated. Student’s t test: * = *p* < 0.05; *** = *p* < 0.001. (**M**,**N**). ELISA showing IL-1β protein levels in the culture media of CD organoids compared to CTR organoids before (NT) and after treatment with P31-43 as indicated. The numbers of organoids analyzed are indicated. The columns represent the mean, and bars represent the standard deviation. Student’s t test: * = *p* < 0.05. (**O**,**P**). ELISA showing IL-6 protein levels in the culture media of CD and CTR organoids before (NT) and after treatment with P31-43 as indicated. The numbers of organoids analyzed are indicated. The columns represent the mean, and bars represent the standard deviation.

Basal differences between patients and controls organoids for the expression of inflammatory markers NF-κB and ERK showed by WB in Figure 3A,C, Figure 4A,D and Figure 5I,L prompted us to study these markers after pro-inflammatory stimuli such as P31-43. CD cells responded to inflammatory stimuli such as the A-gliadin peptide P31-43 by increasing inflammatory markers expression [6]. To determine if there was a difference in sensitivity between CTR and CD organoids, we treated them with P31-43 at concentrations that did not affect CTR organoids (Figure 6).

P31-43 concentrations of 10 μg/mL and 20 μg/mL did not increase the expression of the inflammatory markers pNF-κB, pERK, IL-1β, and IL-6 in control organoids (Figure 6). We analyzed pNF-κB and pERK by WB and IL-1β and IL-6 by quantitative PCR and ELISA (Figure 6).

On the other hand, in CD organoids, the expression of the inflammatory markers pNF-κB and pERK increased when stimulated with P31-43 at 10 μg/mL (pNF-κB *p* value < 0.05, pERK: *p* value < 0.01) and 20 μg/mL (pERK: *p* value < 0.05) (Figure 6A–H).

As measured by quantitative PCR of the total mRNA, IL-1β, and IL-6 (Figure 6I–L) expression increased in CD organoids after both 10 μg/mL (IL-1β: *p* value < 0.0001, IL-6 *p* value < 0.05) and 20 μg/mL (IL-1β: *p* value < 0.0001, IL-6 *p* value < 0.001) P31-43.

ELISAs (Figure 6M–P) on CD organoid culture media revealed that IL-1β expression was increased after 10 μg/mL P31-43 (*p* value < 0.05), whereas IL-6 expression did not increase. These results indicate that CD enterocytes were more sensitive to the inflammatory stimuli of gliadin peptide P31-43 than control enterocytes.

### 2.5. Organoids from CD Patients Were More Sensitive to Lox

CD cells and biopsies responded to the inflammatory stimuli of Toll-like receptor 7 viral ligand, Lox, by increasing the levels of inflammatory markers [9,25]. Considering the basal differences between patients and controls organoids for the expression of inflammatory markers NF-κB and ERK showed by WB in Figure 3A,C, Figure 4A,D and Figure 5I,L, we tested the sensitivity to another pro-inflammatory stimuli such as Lox.

**Figure 7:**
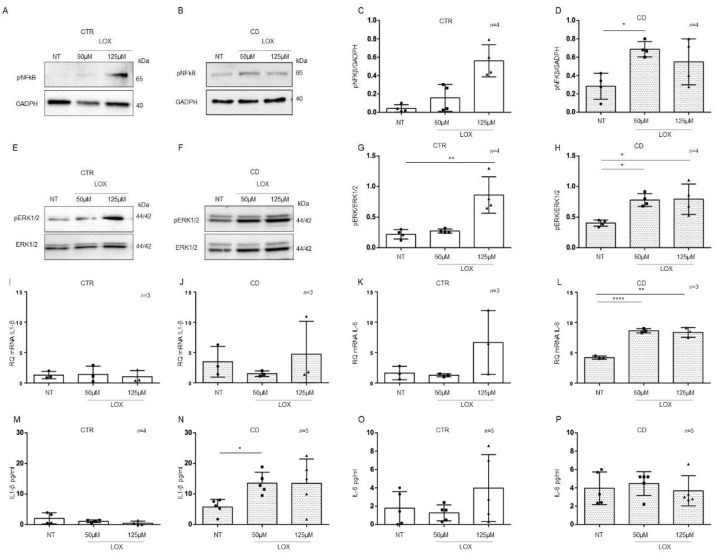
Organoids from CD patients, but not from CTR patients, are inflamed by Lox: Western blot analysis of total protein lysates of organoids from CTR (**A**) and CD (**B**) patients before (NT) and after treatment with low concentrations of Lox (50 μM and 125 μM). Upper lines were blotted with an antibody against the phosphorylated form of NF-κB (pNF-κB). Bottom lines were blotted with anti-GAPDH antibody as a loading control. Representative images were selected. (**C**,**D**). Densitometric analysis of the pNF-κB/GAPDH bands from CTR and CD before and after treatment with Lox as indicated. Columns represent the mean, and bars represent the standard deviation. Student’s *t* test: * = *p* < 0.05. (**E**,**F**). Western blot analysis of total protein lysates of organoids from CTR (**E**) and CD (**F**) patients before (NT) and after treatment with Lox as indicated. Upper lines were blotted with an antibody against the phosphorylated form of ERK (pERK). Bottom lines were blotted with anti-ERK antibody as a loading control. Representative images were selected. (**G**,**H**). Densitometric analysis of the pERK/ERK bands from CTR and CD before and after treatment with Lox as indicated. Columns represent the mean, and bars represent the standard deviation. Student’s *t* test: * = *p* < 0.05; ** = *p* < 0.01. (**I**,**J**). Quantitative PCR analysis of IL-1β mRNA levels in organoids from CD patients compared to those from CTR patients. The numbers of organoids analyzed is indicated. Columns represent the mean, and bars represent the standard deviation. (**K**,**L**). Quantitative PCR analysis of IL-6 mRNA levels in organoids from CD patients compared to those from CTR patients. The numbers of organoids analyzed are indicated. Student’s *t* test. ** = *p* < 0.01; **** = *p* < 0.0001. (**M**,**N**). ELISA showing IL-1β protein levels in the culture media of CD organoids compared to CTR organoids before (NT) and after treatment with Lox as indicated. The numbers of subjects analyzed are indicated. The columns represent the media, and bars represent the standard deviation. Student’s *t*-test: * = *p* < 0.05. (**O**,**P**). ELISA showing IL-6 protein levels in the culture media of CD and CTR organoids before (NT) and after treatment with Lox as indicated. The numbers of organoids analyzed are indicated. The columns represent the media, and bars represent the standard deviation.

To understand if there was a difference in sensitivity between CTR and CD organoids to viral ligand stimuli, we treated them with Lox at concentrations that did not affect CTR organoids (Figure 7).

In control organoids, 50 μM and 125 μM Lox (Figure 7) did not increase the expression of the inflammatory markers pNF-κB, pERK, IL-1β, and IL-6, with the exception of pERK, which was significantly increased only by 125 µM Lox (*p* value < 0.01). As before, we analyzed pNF-κB and pERK by WB and IL-1β and IL-6 by quantitative PCR and ELISA (Figure 7).

On the other hand, in CD organoids, treatment with Lox increased the expression of the inflammatory markers pNF-κB and pERK at both 50 μM (pNF-κB, *p* value < 0.05) and 125 μM (pERK, *p* value < 0.05) concentrations, according to WB analysis (Figure 7A–H).

Stimulation with 50 µM (*p* value < 0.0001) and 125 µM (*p* value < 0.01) Lox increased the levels of IL-6 mRNA (Figure 7K,L) but not IL-1β mRNA.

ELISAs (Figure 7M–P) on CD organoid culture media revealed that IL-1β was increased after 50 µM Lox (*p* value < 0.05) treatment, whereas IL-6 was not increased. These results indicate that CD enterocytes were more sensitive than control enterocytes to inflammatory stimuli of different origins.

## 3. Discussion

The mucosa of the small intestine is the primary target of CD. Gluten peptides interact with the epithelium, cross the epithelial barrier, and induce an adaptive immune response against gluten in individuals with MHC class II DQ2/8 haplotypes. Only a few individuals with these haplotypes will develop CD after exposure to gluten, indicating that other factors influence the initiation and maintenance of the disease. One of these factors could be the altered functionality of the innate epithelial response [20].

In this manuscript, we described epithelial inflammation in CD biopsies using IL-1β and IL-6 as markers in patients at the acute phase of the disease (GCD-CD), after remission of the intestinal lesion (GFD-CD), and in patients with anti-TTG antibodies, who were genetically predisposed to CD (Pot-CD) yet had normal intestinal morphology for 2–3 years (on average) prior to the onset of the intestinal lesion. We derived organoids from intestinal biopsies of GCD-CD and CTR patients and tested them for markers of inflammation, such as pNF-κB, pERK, IL-1β, and IL-6, and found that these markers were altered in CD organoids. In CD organoids the inflammation was persistent. Moreover, CD organoids were more sensitive than controls to inflammatory stimuli such as gliadin peptide P31-43 and the TLR7 receptor ligand Lox.

Intestinal inflammation in CD has been described by many different studies, in patients at both the GCD and GFD stages and before and after gluten challenge [9,11,12,14,17]. It is now clear that in CD, several different factors, such as cellular vulnerability, the proinflammatory effects of gluten and other wheat proteins, Western diet, and other environmental triggers, such as viruses, converge to prepare, and/or amplify the TC-mediated response to gluten [6,9].

Moreover, changes at the mRNA and protein levels of the inflammasome pathway were found in intestinal epithelial cells purified from CD intestinal biopsies and analyzed for gene expression. These results indicate that intestinal epithelial cells play a key role in small intestinal inflammation in CD [20].

The IL-1β and IL-6 cytokines are important mediators of the inflammatory response and are involved in a variety of cellular activities, including cell proliferation, differentiation, and apoptosis [28,32].

In this manuscript, we found that IL-1β and IL-6 levels increased in CD enterocytes, not only in the acute phase of the disease but also in the remission phase and in potential patients before the onset of intestinal disease. Interestingly, in our experiments, both IL-1β and IL-6 levels were altered mainly in the epithelium in all stages of the disease. The presence of inflammation in the Pot-CD group is particularly interesting, as it indicates that inflammation of the epithelium precedes mucosal remodeling, and points to the intestinal epithelium as a key component of the inflammatory response in CD.

The possibility of growing small intestinal organoids has given many researchers a new tool to study the role of the intestinal epithelium in several different diseases [30]. Organoids are a miniaturized and simplified version of an organ produced in vitro in three dimensions with realistic microanatomy. Intestinal organoids are derived from crypt stem cells. Several different groups have used organoids to study CD, revealing the presence of increased staminality, permeability, inflammasome activity, and innate immunity genes with respect to healthy individuals [20,33]. Extracellular matrix (ECM) genes were decreased in CD organoids compared to control individuals [31]. Taken together, these observations indicate that CD intestinal epithelial cells are constitutively different from those in healthy individuals.

In intestinal organoids from IBD, inflammation disappears after 1 week in culture and can be regained only upon INF-αtreatment [29]. Therefore, in IBD, inflammation in intestinal organoids is regarded as a residual effect of the tissue of origin. We derived organoids from the intestinal epithelium of CD patients, IBD patients, and healthy individuals. We confirmed the data on IBD organoids available in the literature. In our experiments, IBD (both Crohn’s and ulcerative colitis) organoids were no longer inflamed after 4 weeks in culture, and both pNF-κB and pERK levels were not different from the control organoids.

In contrast, in CD organoids, we found increased markers of inflammation, such as pNF-κB, pERK, IL-1β, and IL-6, at the protein and mRNA levels. In contrast to that in IBD, inflammation in CD organoids was persistent, as the levels of pNF-κB and pERK did not decrease after more than ten weeks in culture. This suggests that the inflammation in CD organoids is not a residual effect of the tissue of origin but is constitutive.

Possible constitutive alterations in CD, which appear to be independent of the stage of the disease and the gluten content in the diet, have been recently described in vivo in the literature [12,34,35]. Interestingly, several cytokines related to the inflammatory pathway were increased in at-risk CD infants before the onset of the disease and the introduction of gluten to the diet [35].

Moreover, in CD biopsies and fibroblasts, increased sensitivity to inflammatory triggers such as gliadin peptide P31-43, IL-15, and Toll-7-specific viral ligand Lox has been described [18,25,36,37]

For this reason, we treated CD and CTR organoids with two different environmental inflammatory stimuli, gliadin peptide P31-43 and Lox. P31-43 and Lox, ineffective in CTR organoids, were able to induce the activation of NF-κB and ERK and increase IL-1β and IL-6 levels in CD organoids. In CD biopsies and in intestinal organoids, increased sensitivity to inflammatory stimuli from bacteria has been described [19], indicating that intestinal organoids from CD patients are more sensitive to proinflammatory stimuli.

In conclusion, the factors that create a proinflammatory environment in the CD intestine can be exogenous, such as food and viruses, but can also be endogenous. In fact, low-grade inflammation of the CD epithelium, probably constitutive, is present even before intestinal damage. Intestinal organoids reproduced this constitutive inflammation and thus represent a good model for studying epithelial inflammation in CD. Moreover, the intestinal epithelium in CD is more sensitive to proinflammatory stimuli, including gliadin and viruses. Taken together, these observations point to constitutive alterations, probably genetic or epigenetic, which render the CD epithelium more sensitive to inflammatory stimuli such as food components, virus, and microbiota.

## 4. Materials and Methods

### 4.1. Organoids

One to two duodenal biopsies per individual from CD patients and from controls were taken with standard endoscopic EGDS during routine gastroduodenoscopy (Table 1) and placed in ice-cold: 10 mL isolation buffer (5.6 mmol/L Na2HPO4 (Sigma S7907; Sigma-Aldrich, Milan, Italy), 8.0 mmol/L KH2PO4 (Sigma P5655; Sigma-Aldrich, Milan, Italy), 96.2 mmol/L NaCl (Sigma S5886; Sigma-Aldrich, Milan, Italy), 1.6 mmol/L KCl (Sigma P5405; Sigma-Aldrich, Milan, Italy), 43.4 mmol/L sucrose (Fisher BP220-1; Thermo Fisher Scientific, Waltham, MA, USA), and 54.9 mmol/L d-sorbitol (Fisher BP439-500; Thermo Fisher Scientific Milan, Italy)) in deionized water. Crypt units were isolated according to the protocol of Yuli Wang et al. [38] with minor variations. Briefly, after 60 min, the biopsy samples were further enzymatically digested with collagenase (2 mg/mL, C0130 Sigma-Aldrich, Milan, Italy) in washing buffer (WB) containing penicillin/streptomycin (100 units mL^−1^, cat.15140122), l-glutamine (2 mM, cat. 25030081), and FBS (10%, *vol*/*vol*, cat.10270-106) in DMEM/F12 Nutrient Mix (cat.11330-032, Gibco, Milan, Italy) with HEPES (cat.15630-049 Gibco, Milan, Italy) on ice for 30 min. The digest was filtered through a 70 µm strainer (Falcon, NY, USA) and the strainer was rinsed with an additional 10 mL of WB. Crypts were collected by centrifugation at 500× *g* for 5 min. The supernatant was discarded, the crypts were carefully resuspended in 40 µL of ice-cold Matrigel matrix (Corning cat.35623, Milan, Italy) to enable three-dimensional growth in 48-well plates; the plates were incubated in a cell culture incubator at 37 °C and 5% carbon dioxide for 10 min to allow the Matrigel to solidify. Afterwards, 300 µL cell culture medium enriched with supplements (CM-S) was added to each well and was replaced every second day. The organoids were used for assays or cryopreserved at −150 °C. To cryopreserve organoids, they were washed with ice-cold PBS EDTA to remove Matrigel and collected by centrifugation at 500× *g* for 5 min. Organoid pellets were suspended in 1 mL WB, 10% fetal bovine serum (FBS, Gibco, Milan, Italy), and 10% dimethyl sulfoxide, slowly frozen to −80 °C in a cryo freezing container (Nalgene, Sigma, Milan, Italy), and then transferred to −150 °C for long-term storage. For further research, the cryopreserved organoids were quickly thawed at 37 °C, transferred to 10 mL WB, centrifuged at 2000× *g* for 5 min, plated with Matrigel, and cultured in CM-S medium. For 2D organoids, organoids were openly seeded in six wells pretreated with Matrigel diluted 1:40 in phosphate-buffered saline (PBS)

### 4.2. Culture Medium to Maintain Organoids (CM-S)

Mouse l-cells that expressed Wnt3a, R-spondin, and Noggin were commercially purchased (ATCC CRL-3276, Genova, Italy), and a conditioned medium (L-WRN) was prepared according to the instructions and protocol of the manufacturer. Culture medium with supplements (CM-S) was prepared using 50% conditioned L-WRN medium and 50% fresh primary culture media: Advanced DMEM/F-12 (cat.12634-010, Invitrogen Milan, Italy) 1 mM N-Acetyl-l-cysteine (cat. A7250, Sigma, Milan, Italy), 1 × B-27^®^ supplements (cat.12587-010 Gibco, Milan, Italy), 50 ng/mL epidermal growth factor, (cat. PMG8041 Gibco Milan, Italy) 10 mM nicotinamide (cat. N0636, Sigma, Milan, Italy), 10 nM Leu15-gastrin I (cat. G9145 Sigma, Milan, Italy), 500 nM A8301 (inhibitor for ALK4/5/7, cat. 70024-90-7 Sigma, Milan, Italy), 10 µM SB202190 (p38 MAP kinase inhibitor, cat. S7076 Sigma, Milan, Italy), and 10 µM Y-27632 (p160 ROCK inhibitor; cat.1254 Tocris, Milan, Italy) in accordance with the protocols of Sato et al. [39], VanDussen et al. [40] and Yuli Wang et al. [38]. The organoids were cultured with 300 µL culture medium, which was changed every second or third day. After seven days, when the organoids had formed large circular structures, they were isolated from the Matrigel matrix and split. For each passage, the spheroid/Matrigel was scratched into 500 µL of PBS containing 0.5 mM EDTA (PBS-EDTA). The spheroids were pelleted by centrifugation at 500× *g* for 5 min, and the supernatant was discarded. The spheroids were dissociated by incubation in 200 µL of 0.25% trypsin in PBS-EDTA for 60–90 s. An additional 5 mL of WB was then added to inactivate the trypsin. The spheroids were pelleted by centrifugation at 500× *g* for 5 min. The supernatant was carefully removed, and the pellet was resuspended in Matrigel.

### 4.3. Fixing of Organoids, OCT Embedding, and Cryosectioning

After removing the organoid culture media, the human spheroids were washed from each well of a 6-well plate with 5 mL of 1X PBS at room temperature. The structures were fixed with 5 mL of 2% paraformaldehyde (PFA) and 0.1% glutaraldehyde (GA) in 1X PBS for 30 min at room temperature. After washing extensively with 5 mL of 1X PBS to remove the fixing solution, the organoid domes were carefully removed with a scoop or spatula and placed in a 50 mL conical tube containing 20% sucrose in 1X PBS. The tube was left at 4 °C overnight or for three days, until the domes fell to the bottom of the tube. The domes were removed from the sucrose solution and placed in a mold containing optimal cutting temperature (OCT cat. 05-9801 Bio Optica, Milan, Italy) compound. Several domes were placed on each mold; they were snap frozen and stored at −80 °C. Using a cryotome, we cut the organoid block into cryosections approximately 10 µm thick.

### 4.4. Immunostaining

After washing the slides with 1X PBS to remove OCT for 3D organoids, the 2D organoids were openly seeding in six wells pretreated with Matrigel diluted 1:40 in PBS; the tissues were permeated with 0.15% Triton/1X PBS for 15 min at room temperature. The cells were washed 3 times with 1X PBS for 10 min each time. The slides were blocked with 3% BSA/1X PBS (blocking solution) or 10% FBS/1X PBS for 1 h at room temperature. The primary antibody anti-rabbit anti-cytokeratin (cat. Z0622, Dako Carpinteria, CA, USA) was diluted in blocking solution overnight at 4 °C in a humidified chamber. Then the cells were washed 3 times with 1X PBS for 10 min each time. Alexa Fluor 546 donkey anti-rabbit secondary antibody (A10040 Invitrogen, Milan, Italy) was added to the organoids. The cells were then washed 3 times with 1X PBS for 10 min each time, mounted with Mowiol (cat. 475904-M Sigma-Aldrich, Milan, Italy) and observed under a confocal microscope (Zeiss LSM 510, Jena, Germany) [18].

### 4.5. Western Blot

The human spheroids were seeded in six multiwells (Corning, Milan, Italy) coated with Matrigel diluted 1:40 in phosphate buffered saline (PBS) for 3 days. After they were stimulated with P31-43 (Caslo, Kongens Lyngby, Denmark) or LOX (cat. 121288-39-9, Invivogen Toulouse, France) for various times at 37 °C, the organoids were homogenized in tissue homogenization buffer (50 mM Tris–HCl (pH 8), 150 mM NaCl, 5 mM MgCl_2_, 1% Triton, 0.5% sodium deoxycholate, 0.1% SDS, 1 mM PMSF, 1 mM VO_4_, aprotinin, and LAP; all purchased from Sigma, Milan, Italy, except for LAP, which was purchased from Roche, Milan, Italy). The cell lysates were analyzed using SDS–PAGE with a standard running buffer (25 mM Trizma, 192 mM glycine, and 0.1% SDS) and were transferred onto nitrocellulose membranes using Transbolt Turbo (cat.1704158, BioRad, Milan, Italy). The membranes were blocked with 5% nonfat dry milk and probed with rabbit anti-ERK1/2 (cat.31374, Elabscience, Milan, Italy) and rabbit anti-pY-ERK1/2, (cat.20869, Elabscience, Milan, Italy) mouse anti-GAPDH, (cat. G8795, Sigma-Aldrich, Milan, Italy), and rabbit anti-pY-NF-κB, (cat.3033P, Cell Signalling, Euroclone, Milan, Italy). The bands were visualized using ECL (cat. RPN2209, GE Healthcare, Amersham, Buckinghamshire, UK) with exposure times of 2–10 min. The band intensity was evaluated by integrating all the pixels of the band after subtraction of the background to calculate the average of the pixels surrounding the band [18].

### 4.6. ELISA

The levels of IL-1β and IL-6 were measured using commercial test kits (IL-1β cat.850.006.192, IL-6 cat.950.030.192, Diaclone, Besancon Cedex, France) on cell culture supernatants.

### 4.7. RNAscope to Detect IL-1β and IL-6 mRNA

Expression of IL-1β and IL-6 mRNA in situ was analyzed in intestinal biopsies from CD patients at different stages of the disease as well as those from controls. (Table 1) IL-1β and IL-6 mRNA levels were detected by RNAscope™ 2.5 HD Assay—RED (cat.321720, IL-1β 310361, IL-6 cat.310371, ACD–Biotechne, Milan, Italy). Sample preparation, probe hybridization, and signal detection were carried out according to the kit instructions. Positive signals were indicated by dot-shaped red granules in the crypts. Positive and negative probes were used as positive and negative controls. The red-stained cells were counted. Positive staining in more than 10% of the cells was considered positive, while less than 10% or colorless staining was defined as negative.

Briefly, biopsies included in paraffin were cut at 5 μm, slides were deparaffinized with xylene twice for 5 min and 100% alcohol twice for 1 min. Deparaffinized slides were incubated with hydrogen peroxide for 10 min at room temperature, submerged in the target retrieval reagent for 30 min at 99°, transferred to 100% alcohol for 3 min, and dried at room temperature overnight. Each slide was incubated with protease plus for 30 min at 40° and then washed 3 times with distilled water. Slides were incubated with the appropriate probe for 2 h at 40°, washed with wash buffer and incubated with hybridized Amp (from Amp 1 to Amp 6) according to the protocol. To detect the signal, slides were incubated with fast red solution, submerged in staining dishes containing 50% hematoxylin and mounted with EcoMount.

### 4.8. PCR

Total RNA was extracted from organoids using a RNeasy Mini Kit (cat. 74104 Qiagen, Milan, Italy). The mRNA concentration was measured using a Nanodrop spectrophotometer, and the RNA quality was analyzed using agarose gel electrophoresis in Tris/Borate/ethylenediaminetetraacetic acid (EDTA) buffer (TBE, Sigma, Milan, Italy). RNA (1 µg) was reverse transcribed into cDNA using a QuantiTect Reverse Transcription Kit (cat. 205311 Qiagen, Milan, Italy) according to the manufacturer’s protocol. The experiments were performed with approximately 40 ng of cDNA templates, according to the manufacturer’s protocol (cat. 4331182 TaqMan Gene Expression Assay, Applied Biosystems, Monza, Italy), using a 7900 HT Fast Real-Time PCR system. The gene expression assay used to detect the IL-1β gene was Hs01555410_m1 (Applied Biosystems, Thermo Fisher Scientific Inc., Monza, Italy), and the probe was located on Chr.2: 112829758–112836842 on Build GRCh38; for the IL-6 gene Hs00174131_m1 (Applied Biosystems, Thermo Fisher Scientific Inc., Monza, Italy) was used, and the probe was located on Chr.7: 22725889–22732002 on Build GRCh38. The expression of each gene was normalized to the expression of an endogenous housekeeping gene (HPRT1). Relative quantification was performed using the ΔΔCt method. SDS software (ABI, version 1.4 or 2.4) was used to analyze the raw data.

### 4.9. Statical Analysis

GraphPad Prism 5 software (GraphPad Software, San Diego, CA, USA) was used for statistical analyses and to construct graphical representations. Statistical analyses of the differences included Student’s *t* tests. A *p* value < 0.05 was considered statistically significant. Two-tailed comparisons were used for all statistical analyses. The sample size was chosen after considering the variance of the control samples, and the number of samples needed to assess the extent of the expected effect was estimated to be three or four. Therefore, the chosen sample size was three, four, or (more often) five.

## Figures and Tables

**Table 1:** Patient characteristics.

Patients	Range Age (Years)	Sex	Biopsy (Marsh Classification *)	Serum AntiTG2 (U/mL)	Anti-Endomysial Antibody (EMA)
Controls (N =8)	10–18	M = 4, F = 4	8 = T0	0–1.5	Negative
GCD-CD (N = 8)	3–15	M = 3, F = 5	10 = T3 ^c^ 2 = T3 ^c/b^	>50	Positive
GFD-CD (N = 5)	5–18	F = 5	3 = T0 2 = T1	0–1.5	Negative
POT-CD (N = 4)	7–12	M = 2, F = 2	3 = M0 1 = T1	9.3–57	3 = positive 1 = ND

* T0: Normal; T1: infiltrative lesion; T3: Flat destructive lesion (^b^: moderate, ^c^: total).

## Data Availability

Not applicable.

## Supplementary Materials

The following supporting information can be downloaded at: https://www.mdpi.com/article/10.3390/ijms23041973/s1.
