# Supplementary material for: Inflammation Is Present, Persistent and More Sensitive to Proinflammatory Triggers in Celiac Disease Enterocytes
Source: Int J Mol Sci. 2022 Feb 10;23(4):1973. doi: 10.3390/ijms23041973 (PMC8880034; doi:10.3390/ijms23041973)
Supplement: Supplementary file 1 [file ijms-23-01973-s001.zip › ijms-1537204 Supplementary materials- proofed-done.pdf]

International Journal of *Molecular Sciences*

MDPI

Supplementary materials

Inflammation is present, persistent and more sensitive to pro-inflammatory triggers in celiac disease enterocytes

Monia Porpora [1], Mariangela Conte [1], Giuliana Lania [1], Claudia Bellomo [1], Luciano Rapacciuolo [1], Fernando Gabriel Chirdo [2], Renata Auricchio [1], Riccardo Troncone [1], Salvatore Auricchio [1], Maria Vittoria Barone [1,*] and Merlin Nanayakkara [1]

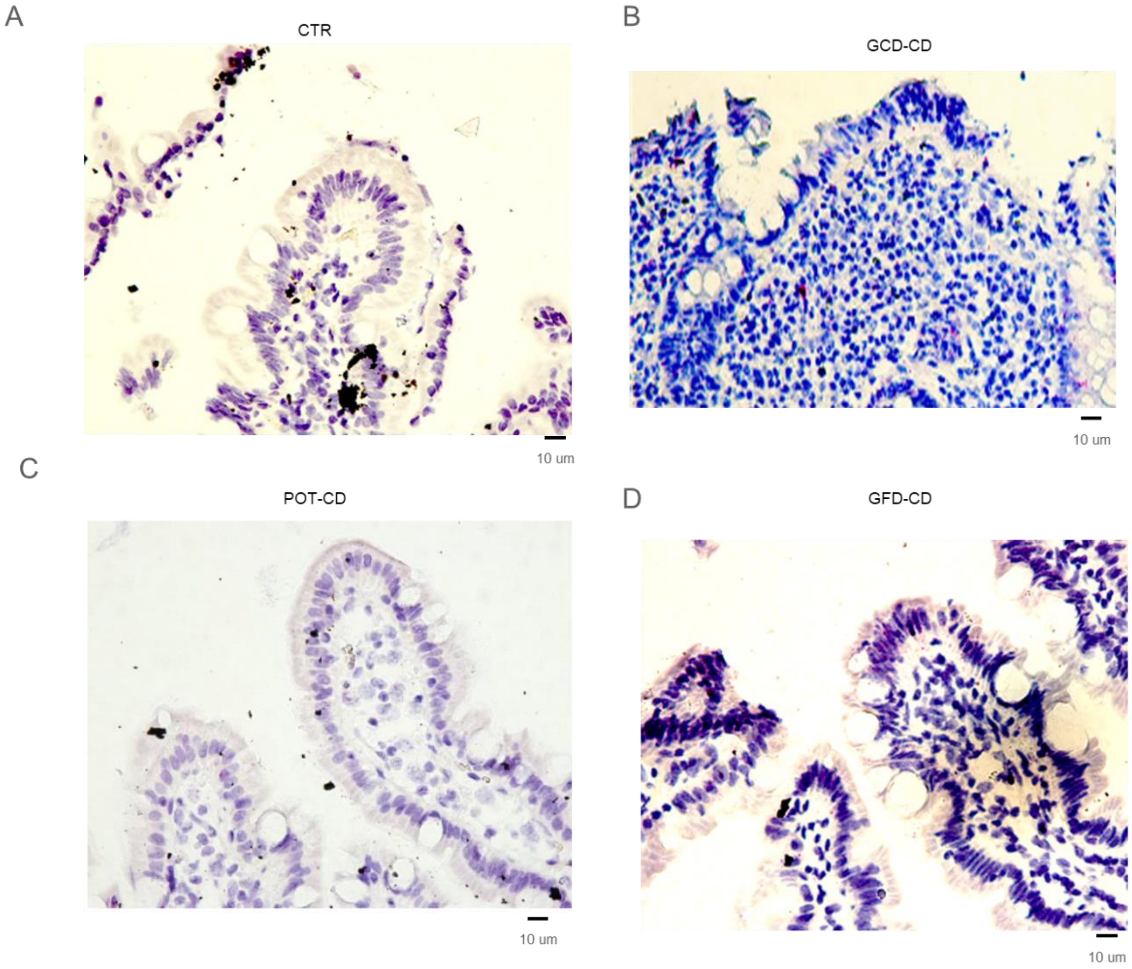

Figure S1. IL-1βis not present in the epithelium of the villi in CD biopsies. In situ mRNA analysis of IL-1βeta in biopsies from controls (CTR) (**A**), gluten-containing diet celiac disease patients (GCD-CD) (**B**), potential CD patients (Pot-CD) (**C**), and gluten-free diet celiac patients (GFD-CD) (**D**). Blue indicates haematoxylin-eosin staining of the nuclei, and red indicates IL-1β mRNA. 40× enlargements. Lines indicate 10 micrometers.

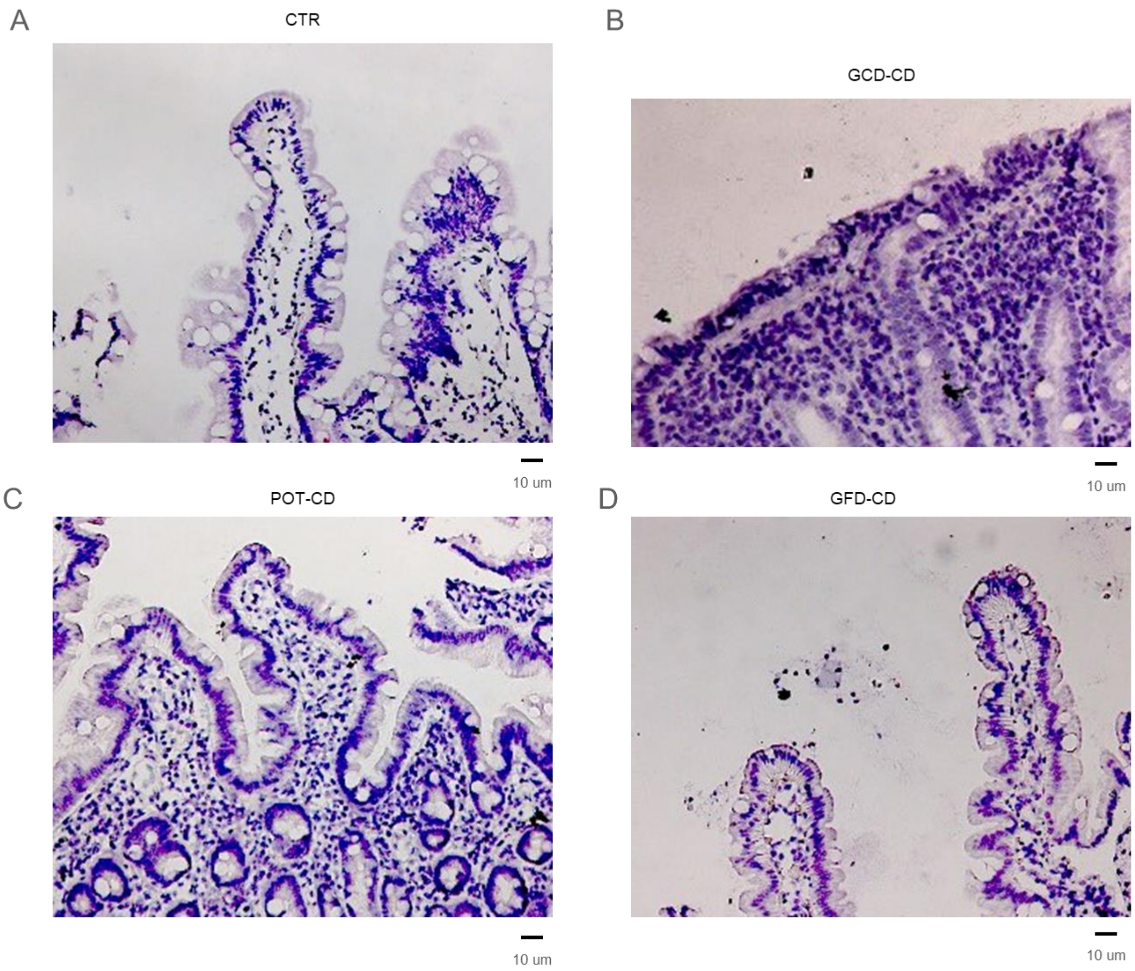

Figure S2. IL-6 is not present in the epithelium of the villi in CD biopsies. In situ mRNA analysis of IL-6 in biopsies from controls (CTR) (**A**), gluten-containing diet celiac disease patients (GCD-CD) (**B**), potential CD patients (Pot-CD) (**C**), and gluten-free diet celiac patients (GFD-CD) (**D**). Blue indicates haematoxylin-eosin staining of the nuclei, and red indicates IL-1β mRNA. 40× enlargements. Lines indicate 10 micrometers.

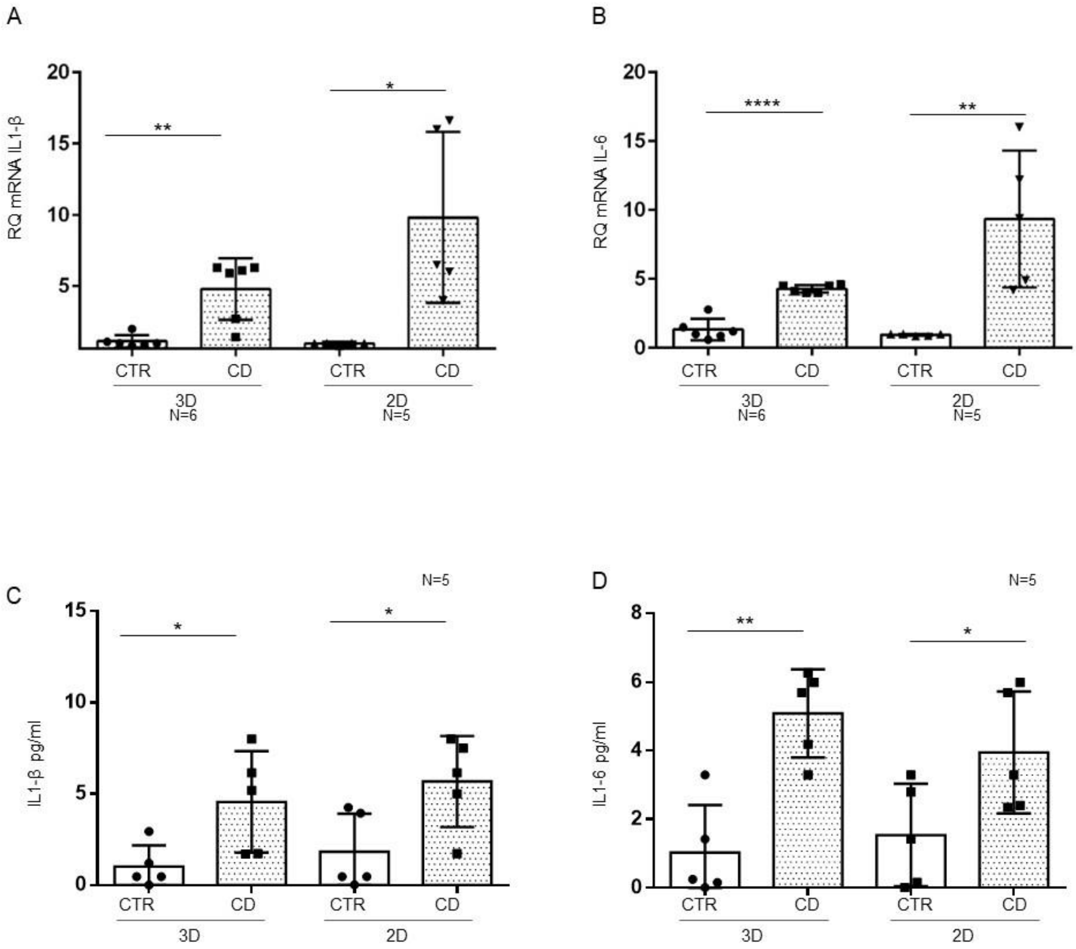

Figure S3. Markers of inflammation were increased in both 3D and 2D organoids from CD patients compared to CTR patients. (**A**,**B**) Quantitative PCR analysis of IL-1β and IL-6 mRNA levels in 3D and 2D organoids from CTR and CD patients. The number of organoids analysed is indicated. The columns represent the mean, and bars represent the standard deviation. Student's *t test*: * = $p < 0.05$; ** = $p < 0.01$; **** = $p < 0.001$ (**C,D**) ELISA showing IL-1β and IL-6 protein levels in the culture media of 3D and 2D organoids from CTR and CD patients. The numbers of organoids are indicated. The columns represent the mean, and bars represent the standard deviation. Student's *t test*: * = $p < 0.05$; ** = $p < 0.01$.
